# Malaria importada en población pediátrica de Alicante, España (1994-2019)

**DOI:** 10.7705/biomedica.6068

**Published:** 2022-06-01

**Authors:** Ana Elena Pascua-Santamaría, Diego Torrús-Tendero, Gema Mira-Perceval-Juan, Paloma García-Galán, José Manuel Ramos-Rincón

**Affiliations:** 1 Servicio de Pediatría, Hospital Marina Baixa, Villajoyosa, Alicante, España Hospital Marina Baixa, Villajoyosa Alicante España; 2 Unidad de Referencia de Enfermedades Importadas y Salud Internacional, Hospital General Universitario de Alicante, Alicante, España Hospital General Universitario de Alicante Alicante España; 3 Unidad de Enfermedades Infecciosas, Hospital General Universitario de Alicante, Alicante, España Hospital General Universitario de Alicante Alicante España; 4 Área de Parasitología, Universidad Miguel Hernández de Elche, Alicante, España Universidad Miguel Hernández de Elche Universidad Miguel Hernández de Elche Alicante Spain; 5 Servicio de Pediatría, Hospital General Universitario de Alicante, Alicante, España Hospital General Universitario de Alicante Alicante España; 6 Servicio de Medicina Interna, Hospital General Universitario de Alicante, Alicante, España Hospital General Universitario de Alicante Alicante España; 7 Departamento de Medicina Clínica, Universidad Miguel Hernández de Elche, Alicante, España Universidad Miguel Hernández de Elche Departamento de Medicina Clínica Universidad Miguel Hernández de Elche Alicante Spain

**Keywords:** malaria, Plasmodium falciparum, niño, España, Malaria, *Plasmodium falciparum*, child, Spain

## Abstract

**Introducción.:**

En los últimos años ha aumentado la malaria importada en niños, en quienes la enfermedad es potencialmente grave y mortal.

**Objetivo.:**

Describir la incidencia y características clínico-epidemiológicas de niños con diagnóstico de conmalaria en Alicante, España, en los últimos años.

**Materiales y métodos.:**

Se hizo un estudio observacional y retrospectivo de los casos de malaria en menores de 15 años, diagnosticados en el Hospital General Universitario de Alicante desde 1994 hasta 2019.

**Resultados.:**

Se registraron 24 casos. El número de casos pasó de dos en el primer quinquenio a 11 en el último. La mediana de edad fue de 6 años (rango intercuartílico: 3 a 8). El 91,6 % procedía de África subsahariana. Los niños nacidos en España de padres inmigrantes que viajan a una zona endémica para visitar a familiares y amigos (*Visiting Friends and Relatives*) representaron el 62,5 % (n=15) y solo consta que recibiera quimioprofilaxis antipalúdica un paciente (6,7 %).

Los signos clínicos más frecuentes fueron fiebre (86,9 %), hepatoesplenomegalia (70,8 %) y anemia (70,8 %). *Plasmodium falciparum* fue la especie más frecuentemente identificada (83,3 %; n=20). El tratamiento más empleado fue la combinación de dihidroartemisina y piperaquina por vía oral (41,6 %, n=10), con evolución favorable en todos los casos.

**Conclusiones.:**

Los casos de paludismo infantil importado están aumentando en los últimos años. Las manifestaciones clínicas son inespecíficas en estos niños, por lo que es importante que los profesionales conozcan la enfermedad y tengan un alto índice de sospecha para iniciar el tratamiento precoz. Además, deben tomarse las medidas preventivas adecuadas antes de un viaje.

## Abstract (en)

**Introduction:**

In recent years there has been an increase in imported malaria among children in whom it is a potentially serious and fatal disease.

**Objective:**

To describe the incidence and the clinical and epidemiological characteristics of malaria in children diagnosed in Alicante, Spain, over a 26-year period.

**Materials and methods:**

We conducted an observational retrospective study of malaria in children aged under 15 years diagnosed at the Alicante General University Hospital from 1994 to 2019.

**Results:**

Twenty-four cases were registered over the study period. The frequency of cases increased from 2 over the first five years to 11 in the last five years. The median age of the children was 6 years (interquartile range: 3-8); 91.6% came from sub-Saharan Africa. Over half (n=15, 62.5%) were children born in Spain to immigrant parents visiting friends and relatives (VFR); just one (6.7%) had received antimalarial chemoprophylaxis.

The most frequent clinical signs were fever (86.9%), hepatosplenomegaly (70.8%), and anemia (70.8%). *Plasmodium falciparum* was the most frequently identified species (83.3%, n=20). The most common treatment was oral piperaquine/dihydroartemisinin (41.6%, n=10) with favorable outcomes in all cases.

**Conclusions:**

Imported childhood malaria shows an increasing incidence and a nonspecific clinical presentation. Professional awareness of this disease and a high degree of clinical suspicion are needed for the early initiation of treatment. Pre-travel preventive measures should be promoted when appropriate.

El paludismo se considera la enfermedad parasitaria de mayor incidencia y mortalidad del mundo. Según el informe de la Organización Mundial de la Salud (OMS), se estima que en el 2018 hubo 228 millones de casos de malaria y 405.000 muertes en el mundo, 67 % de ellas en niños menores de cinco años ^1^.

En 1964, la OMS declaró a España libre de malaria y, desde entonces, los casos notificados han sido todos importados. El aumento de los viajes internacionales, los flujos migratorios y las adopciones, traen como consecuencia el incremento de enfermedades importadas como la malaria. Los datos del Instituto Nacional de Estadística revelan que el número de extranjeros en nuestra provincia aumentó significativamente entre 1998 y el 2007 ^2^. Parte de esta inmigración procede de África, continente en el que se registran la mayoría (93 %) de casos de malaria, según la OMS ^1^.

Algunas series de casos indican que malaria el paludismo es la enfermedad pediátrica importada más frecuente, y los niños representan entre el 15 y el 20 % de todos los casos ^3^^-^^5^. Los niños nacidos en España de padres inmigrantes que viajan a una zona endémica a visitar a familiares y amigos (*Visiting Friends and Relatives*), son los que tienen más riesgo de sufrir malaria grave. En ellos, la malaria cursa con sintomatología inespecífica similar a la de otras enfermedades y su morbimortalidad es alta, sobre todo si no se ofrece el tratamiento precoz ^3^^-^^5^.

En este contexto, el objetivo de este trabajo fue describir las características clínicas, epidemiológicas, diagnósticas y terapéuticas en los niños con diagnóstico de malaria en Alicante.

## Materiales y métodos

Se hizo un estudio observacional, descriptivo y retrospectivo. Se incluyeron niños menores de 15 años con diagnóstico de malaria en el Hospital General Universitario de Alicante desde 1994 hasta 2019. Los casos se obtuvieron del conjunto mínimo básico de datos de los egresos hospitalarios del Hospital. Se revisaron las historias clínicas en papel, utilizando los programas informáticos Orion^®^, Mizar^®^ y Y Abuccasi^®^. La información clínica se anonimizó asignando un código numérico independiente a cada sujeto.

### Diagnóstico parasitológico

El caso de malaria se definió como aquel con prueba microbiológica positiva y cuadro clínico adecuado O correspondientesugestivo. El método diagnóstico en todos los casos fue la visualización de *Plasmodium* spp. en sangre periférica con tinción de Giemsa. En el 2003, se empleó la reacción en cadena de la polimerasa (PCR) por primera vez y la detección de antígenos de *Plasmodium* spp. en sangre por inmunocromatografía (BinaxNow Malaria^®^, Abbott) se ha venido empleando desde el 2006.

Se registraron las variables demográficas (edad, sexo, país de origen, tiempo de estancia en España, país visitado, motivo y época del viaje) y las clínicas (duración de la fiebre hasta el momento del diagnóstico, síntomas y signos en la exploración física), así como los hallazgos relevantes de las pruebas complementarias realizadas, la especie de *Plasmodium* identificada, el índice parasitario, la quimioprofilaxis previa recibida, el tratamiento administrado (fármacos, dosis y duración), la evolución clínica, los pacientes ingresados en la unidad de cuidados intensivos pediátricos, los días desde el ingreso y otras posibles infecciones parasitarias asociadas.

### Análisis estadístico

Los datos se registraron en una base de datos Excel y se analizaron usando el programa estadístico SPSS™, versión 25. Las variables cuantitativas se expresaron en medias y desviaciones estándar (DE) o medianas y rangos intercuatílicos (RIC) en función de si seguían o no una distribución normal. Las variables categóricas se representaron en frecuencias relativas. En caso de requerirse la comparación de variables categóricas, se hicieronzo la prueba de ji al cuadrado y las pruebas no paramétricas si la muestra era muy pequeña. Se fijó la probabilidad máxima de que las diferencias observadas se debieran al azar en un 5 %.

### Consideraciones éticas

El estudio fue aprobado por el Comité de Ética para la Investigación con Medicamentos del Departamento de Salud de Alicante-Hospital General (CEim: PI2020-096).

## Resultados

### Características epidemiológicas

Durante el periodo 1994-2019 se diagnosticaron 24 casos de malaria infanti, apreciándose un aumento de casos en los últimos cinco años. Se evidenció, además, su tendencia a concentrarse en los meses de verano con 10 casos (41,7 %) frente a 2 casos (8 %) en los meses de invierno. En primavera se detectaron 4 casos (16,7 %) y en otoño, 8 (33,3 %).

La mediana de edad fue de 6 años (RIC=3 a 8). El 58,3 % fueron mujeres (14 mujeres frente a 10 varones). El 62,5 % (15 casos) eran hijos de padres inmigrantes que viajaron a su país de origen a visitar a familiares y amigos por un periodo; 6 casos correspondieron a inmigrantes recién llegados a España y, 3 casos, a turistas.

El 91,6 % (22 casos) provenían del África subsahariana, siendo Guinea Ecuatorial con 9 casos (37,5 %) y Nigeria (29,2 %) con 7 casos los países de procedencia más frecuentes. Otros países de origen fueron Camerún y Senegal, con 2 casos (8,3 %) cada uno, seguidos de Gambia, Pakistán, Mali y Ecuador, todos ellos con un caso.

Solo en la historia clínica de 7 pacientes quedó registrado si habían recibido o no quimioprofilaxis antimalárica. De estos, solo uno la había recibido profilaxis, pero no se aclaraba si la había completado correctamente (1 de 7= 14,28 %).

### Características clínicas y de laboratorio

El síntoma principal fue la fiebre (86,9 %) detectado en el momento de la consulta en 20 pacientes, con una duración variable (mediana de 5 días; RIC=2,2 a 8,5). Once pacientes presentaron vómitos (47,8 %), 7 pacientes dolor abdominal (30,4 %), y 5 niños diarrea (21,7 %). Otras manifestaciones fueron escalofríos, referido en 8 niños (34,7 %), tos en 7 (30,4 %), cefalea en 6 (26 %), odinofagia en 5 (21,7 %) y astenia en 5 (21,7 %). En la exploración física, el signo más frecuente fueron las visceromegalias, presentes en 17 pacientes (70,8 %). El 16,6 % (4 niños) presentaba palidez.

Al ingreso, 17 pacientes tenían valores de hemoglobina por debajo de lo normal para su edad ^6^, y se halló trombopenia (<150.000 plaquetas/ mm^3^) en 11 niños. Otra alteración registrada fue la elevación de la proteína C reactiva, con una mediana de 5,4 mg/dl (RIC=2,3 a 8,1), encontrándose el 45,8 % (11 pacientes) por encima de 5 mg/dl (cuadro 1).

**Cuadro 1:** Datos clínicos y analíticos

		n/N
Sintomatología		
	Fiebre	20/23
	Vómitos	11/23
	Dolor abdominal	7/23
	Diarrea	5/23
	Escalofríos	8/23
	Tos	7/23
	Cefalea	6/23
	Astenia	5/23
	Odinofagia	5/23
**Exploración física**		
	Hepatomegalia	3/24
	Esplenomegalia	6/24
	Hepatoesplenomegalia	8/24
	Palidez	4/23
**Datos analíticos**		
	Anemia para su edad	17/24
	Trombopenia (<150.000 plaquetas/mm3)	11/24
	PCR>5 mg/dl	11/22
**Parasitemia inicial (%)**		
	≤1	20/22
	>5	2/22

### Datos microbiológicos

*Plasmodium falciparum* fue la especie más frecuentemente identificada (20 casos, 83,3 %) , seguida de *P. vivax* (2 casos, 8,3 %) . En un caso de los 24 (4,1 %) se identificó infección mixta (*P. falciparum* y *P. ovale*). En el 90,9% (20 casos) consta una parasitemia menor del 1 %. En el 9,1 % (2 casos) se observaron parasitemias elevadas (mayores de 5 %). En los otros dos casos no queda constancia de la parasitemia. (cuadro 1).

De los 21 pacientes diagnosticados desde el 2003, en 14 se empleó la PCR, la cual fue positiva en todos los casos. La detección de antígenos de *Plasmodium* spp. en sangre mediante inmunocromatografía se utilizó a partir del 2006 en 14 pacientes de los 17 diagnosticados desde ese año (14 de 17=82,35 %). El resultado fue positivo en 13 de estos 14 (92,8 %). En 9 pacientes se hicieron controles sucesivos con la prueba de antígenos: en 66,6 % de estos 9 (6 pacientes) permanecía positivo en el séptimo día y, en el 33,3 % (3 pacientes) permanecía positiva en el día 15.

En 18 niños se hizo tamizaje de parasitosis intestinales y todos fueron negativos, excepto uno en quien se detectó *Giardia lamblia* (1 de 18 =5,6%).

### Evolución y tratamiento

El tratamiento más empleado fue la combinación de dihidroartemisina y piperaquina por vía oral en 10 casos. En 6 (6 de 24, 25 %) se prescribió la administración de artesunato intravenoso y en 4 (4 de 24, 16,6 %) tratamiento único. El tratamiento con atovuacona y proguanil se usó en 2 niños . Pudo constatarse en el estudio que el uso de los derivados de artemisininas orales se inició a partir del 2012 y que el artesunato intravenoso se empleó por primera vez en el 2014. El sulfato de quinina en combinación con clindamicina se empleó en 6 pacientes, en 4 por vía oral y en 2 por vía intravenosa. Otros tratamientos usados fueron la cloroquina en 4 casos y la halofrantina en 2 de 1999. La combinación de sulfadoxina y pirimetamina (Fansidar^®^) se prescribió conjuntamente con sulfato de quinina en el 2003 y, con cloroquina, en otro caso de 1995 (cuadro 2).

**Cuadro 2:** Tratamientos administrados

Año	Tratamiento	n
1994-1999	Halofrantina por vía oral	2
	Cloroquina por vía oral	1
	Cloroquina más sulfadoxina-pirimetamina por vía oral	1
2000-2005	Cloroquina por vía oral	2
	Sulfato de quinina más sulfadoxina-pirimetamina por vía oral	1
	Primaquina por vía oral	1
2006-2011	Sulfato de quinina más clindamicina por vía oral	5
2012-2017	Cloroquina más clindamicina más dihidroartemisina-piperaquina por vía oral	1
	Artesunato intravenoso más dihidroartemisina-piperaquina por vía oral	3
	Atovacuona-proguanil por vía oral	1
	Primaquina por vía oral	1
2018-2019	Dihidroartemisina-piperaquina por vía oral	4
	Artesunato intravenoso más dihidroartemisina-piperaquina por vía oral	2
	Atovacuona-proguanil por vía oral	1
	Primaquina por vía oral	1

En 6 pacientes, el tratamiento se complementó con una cefalosporina de tercera generación (cefotaxima o ceftriaxona). Tres pacientes recibieron tratamiento con primaquina durante 14 días, 2 de ellos debido a la infección por *P. vivax* y otro por infección mixta. También, fue necesaria la transfusión de concentrados de hematíes en 5 niños.

Todos los pacientes fueron hospitalizados, con una duración media de 8,2 días, siendo la estancia mínima de tres días y la máxima de 24. De los 24 pacientes, 5 requirieron ingreso en la unidad pediátrica de cuidado intensivo, pero todos evolucionaron favorablemente. No hubo muertes.

### Diferencias clínicas la según la edad

De los pacientes analizados, el 41,6 % (10 niños) eran menores de 5 años y el 58,4 % (14 niños) tenían 5 años o más. Al comparar las características clínicas de ambos grupos, llamó la atención que los mayores de 5 años presentaron síntomas digestivos más frecuentemente que los de menor edad, que en los menores de 5 años hubo más casos de visceromegalias en la exploración y que un porcentaje mayor requirió ingreso en la unidad de cuidados intensivos. Sin embargo, estas diferencias no fueron estadísticamente significativas, en tanto que sí se encontró que fueron más los menores de 5 años que requirieron ingresos de más de una semana (60%, 6 casos *Vs*. 14,3 %, 2 casos. p<0,05) (cuadro 3).

**Cuadro 3:** Comparación entre los niños menores y los mayores de 5 años

	<5 años	≥5 años	p
Fiebre por más de 3 días	5/8	6/12	0,582
Síntomas digestivos	5/9	12/14	0,162
Visceromegalias	9/10	8/14	0,081
Anemia	6/10	11/14	0,324
Trombopenia	5/10	6/14	0,720
Ingreso por más de una semana	6/10	2/14	0,019
Ingreso en la UCIP	3/10	2/14	0,359

UCIP: unidad de cuidados intensivos pediátricos

### Discusión

En los 25 años estudiados, se diagnosticaron en el Hospital General Universitario de Alicante 24 casos de malaria en menores de 15 años. Al igual que en otros estudios en España y Europa ^7^^-^^15^, en este se constató un incremento de los casos de malaria infantil en los últimos años.

Todos los casos diagnosticados eran importados. correspondía a s La mayoría correspondía a familias de África subsahariana, sobre todo de Guinea Ecuatorial y Nigeria, que fueron a visitar a familiares y amigos. A pesar de que gran parte de los inmigrantes de Alicante procede de países de Latinoamérica, solo se describió un caso procedente de esta área (de Ecuador en el 2013), lo que refleja la reducción de la malaria en Centroamérica y Suramérica entre el 2005 y el 2014 ^16^. Vale la pena mencionar también a Pakistán, país endémico de malaria, no solo por *P. vivax*, como el registrado aquí, sino también por *P. falciparum*^17^.

La mayoría de los viajes se desarrollaron en periodo estival (de junio a septiembre), lo que coincide con las vacaciones de los niños y, en muchos casos, las de los padres. Esta época es la temporada de lluvias en África subsahariana y, por lo tanto, el periodo de máxima incidencia de malaria. A diferencia de la población adulta, en la que se ha visto un aumento relacionado con el turismo, en el caso del paludismo infantil la mayoría de los casos corresponde a hijos de inmigrantes que han nacido o llevan años viviendo en España y viajan al país de origen de la familia para pasar una temporada durante las vacaciones estivales para visitar a familiares y amigos.

Debe destacarse que, según los datos disponibles, solo un niño recibió quimioprofilaxis. Esta ausencia de una apropiada quimioprofilaxis en los niños que viajan de una zona no endémica de malaria a una endémica, se refleja también en otros estudios ^9^^-^^14^. La falta de quimioprofilaxis puede deberse tanto a la falsa creencia de los padres de la existencia de una inmunidad innata contra *Plasmodium* spp., por lo que no consultan para conocer las medidas preventivas necesarias, como al desconocimiento de los profesionales sanitarios. En este sentido, tiene especial importancia el médico pediatra de atención primaria, quien debe tomar consciencia del potencial riesgo de estos niños frente a la malaria. El médico debe proporcionar a los padres información sobre la enfermedad, explicarles el uso de las medidas de barrera, como los repelentes y las mosquiteras, así como indicar la quimioprofilaxis antipalúdica adecuada.

La clínica que presentan los niños puede ser variable e inespecífica. En nuestro estudio, casi el 87 % (20 casos) presentó fiebre en el momento del diagnóstico, hallazgo similar al de otros estudios ^9^^-^^14^, de ahí que siga vigente la máxima de “todo niño con fiebre procedente de un país tropical tiene malaria hasta que se demuestre lo contrario”.

Tras infecciones repetidas, los niños procedentes de un área endémica de malaria pueden desarrollar una inmunidad parcial, es decir, no están protegidos completamente, pero presentan infecciones con parasitemias bajas, son asintomáticos, no presentan fiebre, o tienen síntomas leves. En un estudio en 24 hospitales de Madrid ^10^, se comparó el cuadro clínico de los niños con malaria entre aquellos que viajaron a visitar a familiares y amigos y aquellos que no lo hicieron, y se evidenció que los que sí viajaron presentaban con más frecuencia fiebre (98 % *Vs*. 69 %). Sin embargo, en nuestro estudio, de los 3 niños que no presentaron fiebre, lo era dos habían viajado a su lugar de origen y otro no había viajado. La frecuencia de síntomas gastrointestinales en los niños coincidió con lo reflejado en otros estudios ^9^^,^^10^. Otras manifestaciones clínicas también descritas en la literatura especializada son losescalofríos, la tos, la cefalea, la odinofagia y laastenia. Dada esta sintomatología tan inespecífica, es necesario que el pediatra conozca la enfermedad y tenga un alto índice de sospecha, para solicitar las pruebas diagnósticas e iniciar el tratamiento lo más tempranamente posible.

En la exploración física se encontraron visceromegalias y, a nivel analítico, anemia, en más del 70 % (17 pacientes). A partir de lo ya descrito, puede concluirse que la triada encontrada en nuestros pacientes fue fiebre, visceromegalias y anemia. Ante este cuadro, en el diagnóstico diferencial hay que incluir la leishmaniasis visceral, frecuente en nuestra zona mediterránea.

*Plasmodium falciparum* fue la especie más frecuente, en la mayoría de los casos con una parasitemia baja. Para su detección, tal como lo recomiendan las guías, se usó en todos los casos la visualización del protozoo en sangre periférica, evidenciándose una sensibilidad del 95,8 % en el primer análisis. La detección mediante amplificación de ácidos nucleicos se usó desde el 2003, con una sensibilidad en nuestro estudio del 100 %. Se recomienda cuando esté disponible, ya que permite confirmar la especie y detectar parasitemias mixtas que podrían pasar desapercibidas en el microscopio. La detección del antígeno con inmunocromatografía se empezó a hacer desde el 2006 en la mayoría de los pacientes, con una sensibilidad superior al 90 %, pero más de la mitad de ellos permanecía positivo una semana después, por lo que no es útil para evaluar la reacción terapéutica.

El manejo de la malaria infantil ha supuesto un desafío para los profesionales que atienden a estos pacientes. Por un lado, las guías se basan en la experiencia en las zonas endémicas y no son específicas para la malaria pediátrica importada. Además, hasta hace poco, había escasos estudios sobre la eficacia y seguridad de los fármacos antimaláricos en niños y no existían formas de presentación adecuadas para su administración a los más pequeños. En el presente estudio, se observó un cambio en la terapéutica con la introducción de los derivados de artemisininas, lo que va en consonancia con las últimas recomendaciones.

Desde el 2012, la combinación de dihidroartemisina y piperaquina por vía oral fue el tratamiento de elección, en muchas ocasiones tras la administración de artesunato intravenoso, el cual debe ser la primera línea en casos de malaria grave. Antes del uso de los derivados de artemisininas, el tratamiento más usado era el sulfato de quinina en combinación con clindamicina. La doxiciclina, comúnmente administrada en adultos en combinación con quinina, no se empleó en ninguno de los niños. Cabe recordar que está contraindicada en menores de 8 años por sus efectos secundarios sobre el cartílago de crecimiento y la dentición. Se usó primaquina cuando se identificó *P. vivax* o *P. ovale*, después de determinar los niveles de glucosa-6-fosfato deshidrogenasa la glucosa-6-fosfato deshidrogenasa (G6PDH).

La duración media de la hospitalización fue de ocho días y solo en dos casos, reportados en 1994 y 1995, la duración fue mayor de dos semanas. En la reducción de la estancia hospitalaria puede influir el inicio de las terapias combinadas que incluyen derivados de la artemisinina, el diagnóstico más rápido por disponer lde la prueba de antígenos y la PCR, así como el aprendizaje de los facultativos sobre el manejo clínico de la enfermedad.

Según los datos de la OMS, los niños menores de 5 años son los más vulnerables. En la presente serie, este grupo representó casi el 42 % (10 niños), resultados similares a los de otras publicaciones españolas ^9^^,^^10^. En nuestro este estudio no se encontraron diferencias estadísticamente significativas en la forma de presentación clínica ni en los análisis, tampoco en la necesidad de ingreso en la unidad pediátrica de cudado intensivo, probablemente por el bajo número de casos, pero sí en cuanto a los menores de 5 años que requirieron hospitalizaciones más prolongadas que los mayores de 5 años.

Entre las limitaciones del estudio, cabe señalar que se trató de un estudio retrospectivo, y los datos obtenidos dependían de su adecuado registro por parte del personal sanitario y de su completitud. Algunos datos, como los referentes a las pruebas diagnósticas, no se pudieron consultar en los programas de laboratorio y solo se obtuvo la información reflejada en las historias clínicas. Aunque se recomienda la hospitalización del niño por la posibilidad de complicaciones, es posible que en casos muy leves se haya administrado tratamiento ambulatorio, lo que implicó perderlos para el estudio, ya que la fuente de búsqueda fue únicamente el conjunto mínimo básico de datos.

Al igual que en los adultos, la malaria en la población infantil va en aumento como consecuencia, sobre todo, de la inmigración procedente del África subsahariana, siendo los niños hijos de personas que fueron a visitar a familiares y amigos el grupo de riesgo más importante. Ante las manifestaciones clínicas inespecíficas de los casos pediátricos, es importante tener un alto índice de sospecha para el diagnóstico y el tratamiento precoces. Además, debe hacerse hincapié en la profilaxis y otras medidas preventivas, siendo los pediatras de atención primaria un pilar fundamental para que estas se lleven a cabo.
